# Calculation Method for the Cracking Resistance and Bearing Performance of SFRAC Beams

**DOI:** 10.3390/ma16134769

**Published:** 2023-07-01

**Authors:** Qian Zhu, Jie Liu

**Affiliations:** 1 School of Civil Engineering and Architecture, Zhengzhou University of Aeronautics, Zhengzhou 450046, China; 2 School of Water Conservancy and Environment, Zhengzhou University, Zhengzhou 450001, China; 3 China Construction Seventh Engineering Bureau Co., Ltd., Zhengzhou 450004, China

**Keywords:** SFRAC, replacement rate of recycled aggregate, steel fiber volume fraction, cracking, flexural performance

## Abstract

The utilization of recycled aggregate (RCA) in preparing recycled concrete (RAC) is an effective measure to solve the increase in construction waste. Furthermore, applying RAC to flexural members is a viable practice. The addition of steel fiber to RAC to prepare steel fiber recycled concrete (SFRAC) solves the performance deterioration caused by the recycled aggregate, so it is necessary to study the effects of the recycled aggregate replacement rate and fiber–volume ratio on the crack resistance and bending performance of SFRAC beams. In this study, 13 beams were designed and manufactured, with the water–cement ratio, recycled aggregate replacement rate, and fiber–volume ratio as the primary variables, and the cracking moment and ultimate moment of the SFRAC beams were systematically studied. The results show that the cracking and ultimate moments of the SFRAC beams increased with decreases in the water–cement ratio or with increases in the fiber–volume ratio and were unaffected by the replacement rate of recycled aggregates. Based on the experimental results and theoretical analysis, a calculation model and formula for the cracking moment were established. The ultimate bearing capacity of SFRAC beams can be accurately determined using the ACI 318 and ACI 544 standards. The research results serve as a valuable reference for the design of SFRAC beams, effectively address the issue of performance degradation in RAC structural members, and promote the use of green building materials.

## 1. Introduction

Concrete, as the primary building material in the construction industry, is experiencing rapid growth worldwide. The annual consumption of concrete is estimated to exceed 10 billion cubic meters [1], leading to a shortage of natural resources like sand and gravel. The over-exploitation of these natural resources and resulting environmental damage have become critical and increasingly severe problems that must be solved urgently [2]. At the same time, the demolition of buildings, bridges, and hydraulic structures generates a substantial amount of construction waste, constituting 50% of the total urban waste [3]. Sustainable development requires the use of renewable resources and reducing the consumption of primary natural resources [4]. Transforming waste concrete into recycled aggregate (RCA) is recognized as an effective way to achieve sustainable development in the construction industry [5].

Due to the presence of microcracks and holes, recycled aggregate concrete (RAC) exhibits weaker performance compared to ordinary concrete [6]. Recycled concrete beams have lower bearing capacity [7], larger crack width [8], smaller crack spacing [9], lower stiffness [8,10], greater deflection under both short-term load [11] and long-term load [12,13,14,15,16], and shorter fatigue life [17] when compared to ordinary concrete beams. As a result, recycled concrete is extensively applied in roadbeds and pavements [18,19], while its use in load-bearing structures remains limited, thus constraining the potential utilization of recycled aggregates [20].

The incorporation of steel fibers into concrete has been found to improve its strength, toughness, and crack resistance. This enhancement leads to overall improvements in concrete’s strength and deformation performance [20,21,22,23]. The addition of steel fibers also enhances the mechanical properties of the recycled concrete and produces steel fiber recycled concrete (SFRAC). Ali Babar [21] found that the mechanical properties of concrete with 50% RCA and 0.5% GF, such as compression strength, split tensile, and bending strength, are better than those of ordinary natural aggregate concrete. The influence of RCA and GF contents on the permeability coefficient can be neglected. The addition of steel fiber to recycled concrete beams has enhanced their flexural performance [22,23], reducing crack width and protecting steel bars from corrosion [24]. After the addition of steel fiber to recycled concrete, the mechanical properties and reliability of SFRAC beams compare favorably with those of ordinary concrete beams [25]. They demonstrate improved bearing capacity, rigidity, crack resistance, and reduced deflection [25,26]. Moreover, the cost of recycled aggregates is 2.5 times that of those with added steel fiber [20]. The overall cost of SFRAC beams is lower than that of ordinary concrete beams under the same conditions. Furthermore, SFRAC beams have better bearing capacity, displacement, strain, cracking, and flexural properties than ordinary natural aggregate concrete beams [27].

A literature review showed that SFRAC has good bending performance [23]. The reliability of SFRAC beams is the same as that of ordinary concrete [25]. Although SFRAC beams have been extensively researched, the impact of the water–cement ratio, recycled aggregate content, and steel fiber volume fraction on their flexural capacity and the cracking moment is yet to be thoroughly examined. This study presents a calculation model and formula for the cracking moment and ultimate bearing capacity of SFRAC beams with normal sections. In this study, 13 SFRAC beams were designed and manufactured. The effects of the water–cement ratio, recycled aggregate replacement rate, and steel fiber–volume ratio on the cracking moment and flexural capacity were evaluated. The calculation model and formula for the normal-section bearing capacity and cracking moment of SFRAC beams considering the effects of the fiber content and recycled aggregate replacement rate were established. The feasibility of using ACI318 and ACI544 to calculate the bearing capacity of the normal section was verified.

## 2. Experimental Program

This study mainly investigates the effects of the following parameters on the flexural behavior of concrete beams:

(i) Water–cement ratio: Recycled concrete with three water binder ratios of 0.55, 0.4, and 0.3 was studied.
(ii) Recycled aggregate replacement rate (by a mass fraction): Four recycled aggregate replacement rates of 0, 30%, 50%, and 100% were studied.
(iii) Steel fiber volume fraction: Five different steel fiber contents of 0, 0.5%, 1.0%, 1.5%, and 2.0% were adopted.

The concrete cube compression, axial compression, and splitting tensile strength standard tests were carried out to examine the effects of water–cement ratio, recycled aggregate replacement rate, and fiber volume fraction on compressive strength and tensile strength.

### 2.1. Materials and Mixture Proportions

**Figure 1:**
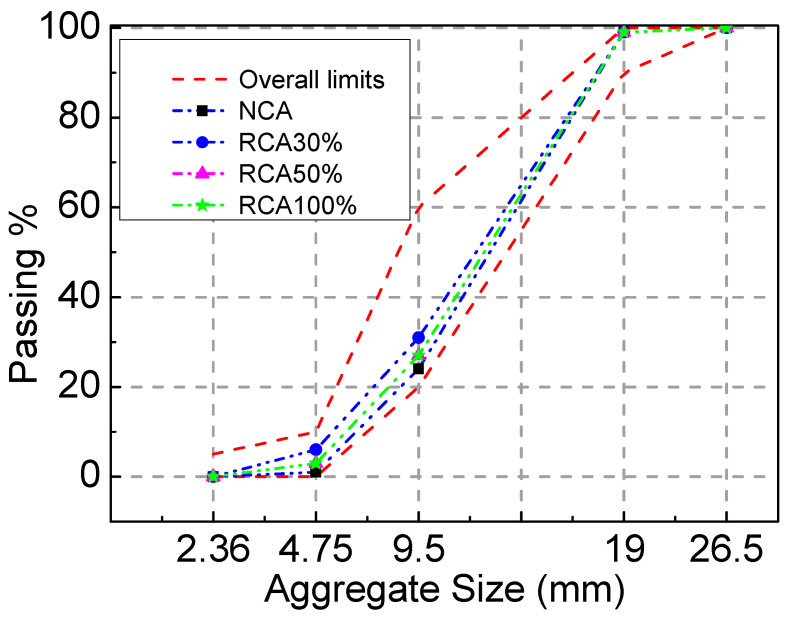
Particle size distribution of coarse aggregates.

**Figure 2:**
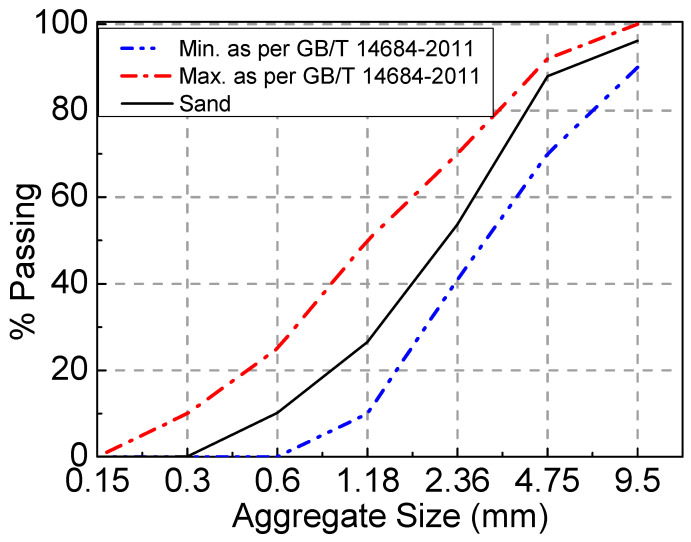
Particle size distribution of fine aggregates.

Common Portland cement P.O 42.5 was used in the experiment, and its performance meets the GB 175-2007 [28] requirements. The detailed properties of cement are presented in Table 1. The nature coarse aggregate (NCA) was crushed limestone with a particle size of 5~20 mm, and its properties conformed to the stipulations in GB/T 14685–2011 [29]. The recycled coarse aggregate was the waste concrete beam with a strength grade of C40 that was crushed using a jaw crusher and sieved to keep the particle size between 5 mm and 20 mm. The detailed properties of the NCA and RCA are presented in Table 2. Both the NCA and RCA had continuous gradation. As illustrated in Figure 1, the particle–size distribution of coarse aggregates satisfied the limitation in GB/T 14685–2011 [29]. The fine aggregate was river sand with a fineness modulus of 2.75. As illustrated in Figure 2, the particle–size distribution of fine aggregates satisfied the limitation in GB/T 14685–2011 [29]. A superplasticizer with a water-reducing ratio of 25% was used, and its optimum dosage was 1% of the cement weight. The water was tap water. The steel fiber that was hooked at both ends had a tensile strength ≥ 1345 N/mm^2^, average length (*l*_f_) of 35 mm, mean diameter (*d*_f_) of 0.55 mm, and aspect ratio (*l*_f_/*d*_f_) of 63. The performance indexes of the longitudinal tensile steel bar and stirrups of the test beam were measured according to GB/T 228.1-2010. The ultimate strength, yield strength, and elongation of the steel bars with a diameter of 16 mm were 623 MPa, 483 MPa, and 24%, respectively. The ultimate strength and elongation of the steel bars with a diameter of 8 mm were 493 MPa and 41%, respectively.

As shown in Table 3, a total of 13 concrete mix ratios with different water–cement ratios, recycled aggregate replacement rates, and steel fiber volume fractions were prepared. The target strengths of concrete with water–cement ratios of 0.55, 0.4, and 0.3 were 30 MPa, 45 MPa, and 60 MPa, respectively. The SFRAC mix ratio and beam measurement results are shown in Table 3. The number after “C” in the beam test piece number represents the water–cement ratio, and 30, 45, and 60 correspond to 0.55, 0.4, and 0.3, respectively. The number after the water–cement ratio “R” represents the recycled aggregate replacement ratio. The number after “F” represents the steel fiber volume fraction. For example, C45R50F1 indicates that the water–cement ratio of the test piece is 0.4, the recycled aggregate replacement ratio is 50%, and the steel fiber volume fraction is 1%. Due to the large water absorption rate of the recycled aggregate, the pre-wet water consumption was calculated based on the water absorption rate of the saturated surface. After pre-wetting for 30 min, SFRAC was mixed with a forced mixer [30]. The mechanical properties of SFRAC are shown in Table 4.

### 2.2. Dimensional and Geometrical Properties of the Specimens

**Figure 3:**
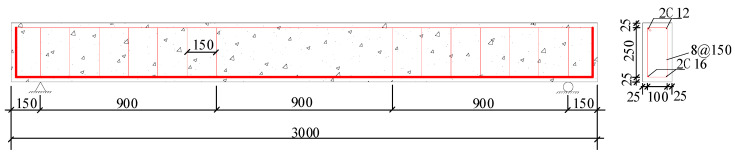
Reinforcement of test beam (unit: mm).

Thirteen reinforced SFRAC beams were made, all of which had a rectangular section of 30 cm × 20 cm (height × width), a length of 300 cm, and a calculated span of 270 cm. The reinforcing bars are shown in Figure 3. The width and height of the beams were 150 mm and 300 mm, respectively. The diameter of the stirrup was 8 mm. Two 12 longitudinal bars were at the bottom, and two 12 steel bars were at the top. The steel bar arrangement is described in Figure 3. The concrete protection layer was 25 mm thick. Each beam was accompanied by three cube compression blocks and three split tensile crack resistance test blocks to measure the concrete compression and the tensile strength.

### 2.3. Test Equipment

**Figure 4:**
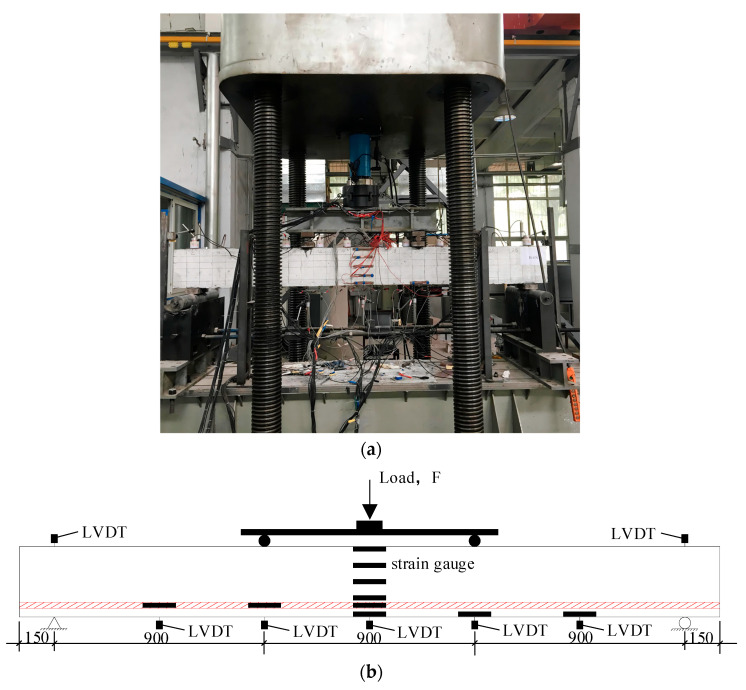
Loading and measuring of test beam. (**a**) Loading photo. (**b**) Schematic of flexural test setup and locations of LVDTs.

All beams were loaded at four points to failure by the distribution beam on the universal testing machine. The test was carried out according to the loading method and procedure of the *Concrete Structure Test Method Standard* (GB/T50152-2012) [31]. Before the cracking load, the load was increased by 2.5 kN per level. After cracking, the load was increased by 5 kN per level until the yield load. The load was continuously increased, and the data were collected until the beam specimen was destroyed. Ten YWC-100 resistive displacement sensors were used to monitor the deformation. The loading method and the measurement arrangement are described in Figure 4. Five concrete strain gauges 100 mm long were evenly arranged from top to bottom in the middle of the span. A steel strain gauge 5 mm long was fixed at the main reinforcing bar span in the longitudinal direction. The schematic diagram of the equipment is shown in Figure 4.

## 3. Test Results and Analysis

### 3.1. Test Phenomenon and Failure Mode

**Figure 5:**
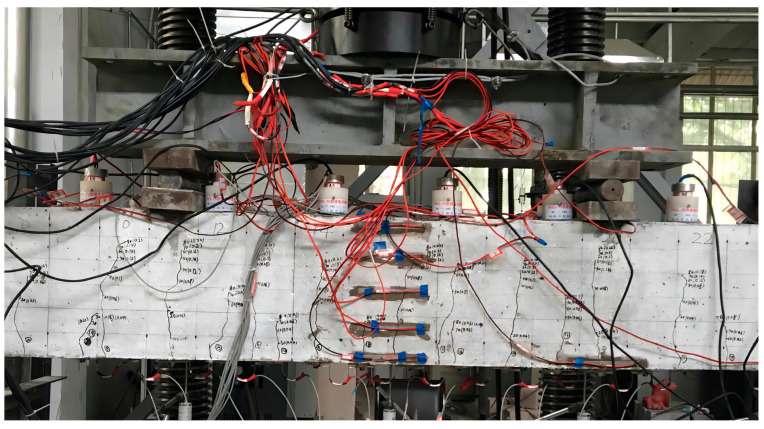
Damage photo of beam C45R50F0.5.

**Figure 6:**
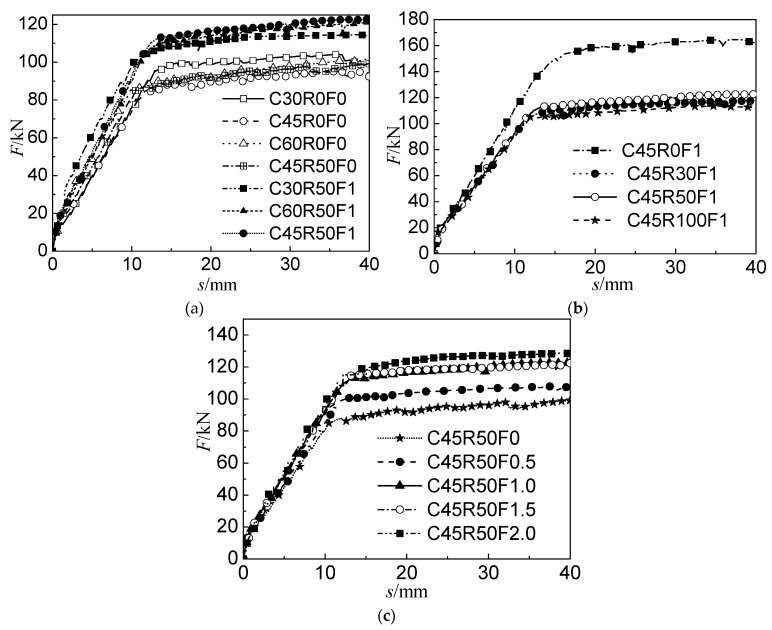
Load versus mid-span deflection. (**a**) Beams with different water–binder ratios. (**b**) Beams with different replacement ratios. (**c**) Beams with different steel fiber volume fractions.

All beams were gradually and evenly loaded to failure. The damage photo of beam C45R50F0.5 is shown in Figure 5. The crack development of each beam under the load at each level was tracked and marked. The cracks and final load are shown in Table 4 [32]. After the static load was gradually applied, the first cracks of all beams were observed in the pure bend section. Before the first crack appeared, all beams showed steep linear elastic behaviors. As the load increased, the crack extended upward, other bending cracks continued to appear along the longitudinal direction of the beam, and the deformation increased. Most bending cracks developed in the vertical direction, and the bending shear cracks appeared. The load was applied continuously, and the longitudinal tensile bar was yielded. Finally, the concrete was crushed, and the beam failed. After each beam failed, the failure mode was recorded. The load–deflection curve of the beam specimen is shown in Figure 6. The load increased slightly from the yield of the tensile steel to the final crushing of SFRAC, while the deflection increased significantly.

### 3.2. Effects of Water–Cement Ratio

**Figure 7:**
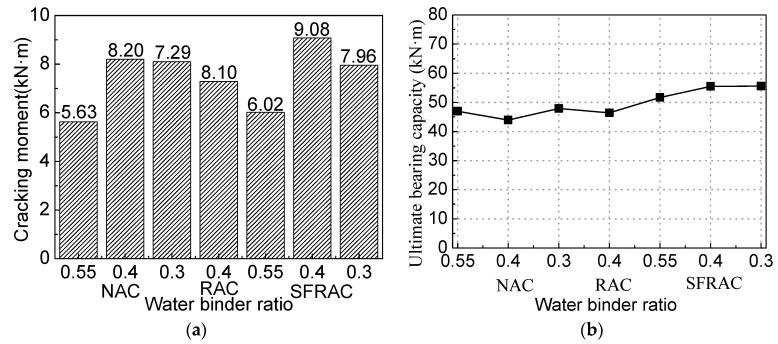
Comparison of test results for beams with different replacement ratios of RCA. (**a**) Cracking moment. (**b**) Ultimate bearing capacity.

According to the test results in Table 4, the effects of the water–cement ratio on the cracking moment of the beam are shown in Figure 7a. The cracking moment of the RAC beam is lower than that of the ordinary concrete beam with the same water–cement ratio, which is consistent with previous studies [8]. However, when the recycled concrete is added to the steel fiber, its cracking moment is greater than that of the ordinary concrete beam with the same water–cement ratio. As the water–cement ratio decreases, the cracking moment of the SFRAC beam increases. The decrease in the water–cement ratio decreases the internal porosity of the recycled concrete, increases the effective cross-sectional area, and enhances the tensile capacity of SFRAC.

According to the test results in Table 4 and the effects of the water–cement ratio on the flexural capacity of the beam shown in Figure 7b, the flexural capacity of the recycled concrete beam is basically the same as that of the ordinary concrete beam with the same water–cement ratio, which is consistent with the conclusions of other scholars [8]. The flexural capacity of the SFRAC beam is larger than that of the ordinary concrete beam and the recycled concrete beam with the same water–cement ratio, and it increases with the decrease in the water–cement ratio. After the concrete at the tensile part of the SFRAC beam is cracked, the steel fiber spanning the crack can bear partial tensile force, making its ultimate bending moment larger than that of ordinary concrete beams and recycled concrete beams. The smaller the water–cement ratio, the higher the compressive strength of the SFRAC, the greater the bond between the recycled concrete at the cross-section tensile part and the steel fibers, and the larger the ultimate bending moment.

### 3.3. Effects of the Recycled Aggregate Replacement Rate

**Figure 8:**
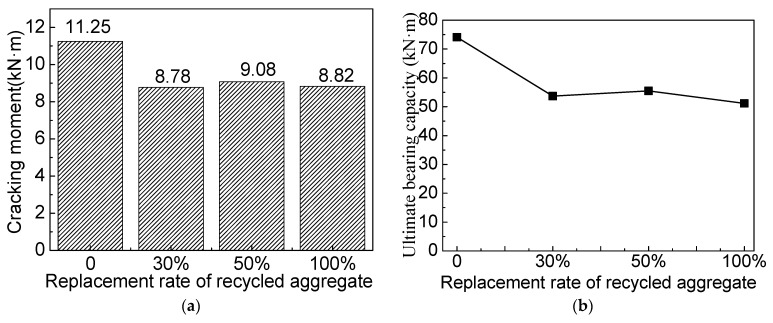

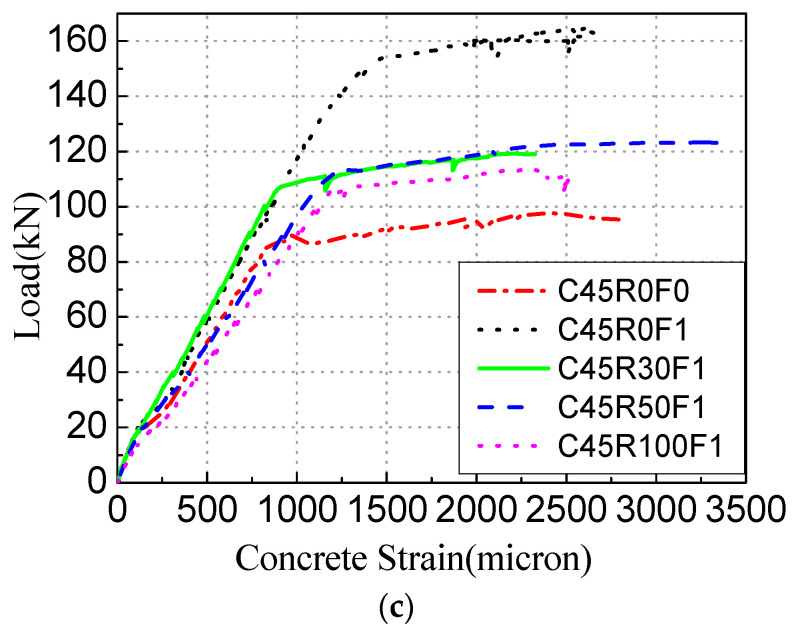
Comparison of test results for beams with different water–binder ratios. (**a**) Cracking moment. (**b**) Ultimate bearing capacity. (**c**) Concrete strain.

The cracking moment of the RAC beam decreases with the increasing replacement rate of the recycled aggregate [8]. As shown in Figure 8, the cracking and ultimate bending moments of the SFRAC beam are smaller than that of the SFNAC beam. However, the RCA change has little influence on the cracking moment and the ultimate bearing capacity of the SFRAC beam, which is consistent with the conclusions of Wael [33]. The SFRAC beam has lower cracking and ultimate bending moments than the steel fiber concrete beam with the same water–cement ratio, as shown in Figure 8. When RCA replaces NCA, the cracking moment and the ultimate bearing capacity decrease. When the RCA replacement rates are 30%, 50%, and 100%, the cracking moments of the SFRAC beam are reduced by 22%, 19.32%, and 21.56%, respectively, and the ultimate bearing capacities of the SFRAC beam are decreased by 27.6%, 25.1%, and 31.0%, respectively. Compared with the NAC reference beam with the steel fiber volume fraction of 0%, when the RCA replacement rates are 30%, 50%, and 100%, the cracking moments of the SFRAC beam are increased by 7.14%, 10.99%, and 7.69%, respectively. The ultimate bending moments of the SFRAC beam are increased by 22.23%, 26.33%, and 16.50%, respectively.

The basic mechanical properties test of SFRAC and the bond performance test of the steel bar and SFRAC demonstrate that the compressive strength of SFRAC and the bond strength of the steel bar and SFRAC decrease with the increasing replacement rate of recycled aggregate. When the ultimate bending state is reached, the bond strength between the steel fiber and the concrete spanning the crack in the tensile region of the SFRAC beam decreases with the increasing replacement ratio of the recycled aggregate, and the tensile force of the steel fiber decreases. Meanwhile, the compressive strength of concrete at the compression part of the beam also decreases, making the ultimate bearing capacity of the SFRAC beam lower than that of the steel fiber ordinary concrete beam. Figure 8c presents the RC beam load concrete stress–strain relationship with the recycled aggregate replacement rate of 50% and different steel fiber volume fractions. Adding steel fiber can enhance the ultimate compressive strain corresponding to the ultimate load and increase the strain when the recycled concrete is crushed from 0.0028 (no steel fiber) to 0.0046 (steel fiber volume fraction 2%), which is consistent with a study conducted by Oh [34].

### 3.4. Effects of the Steel Fiber Volume Fraction

**Figure 9:**
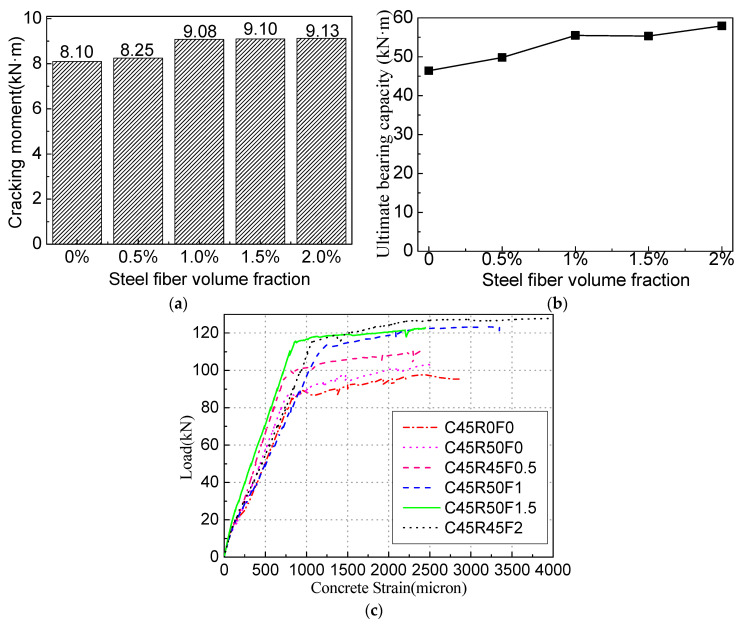
Comparison of test results for beams with different steel fiber volume fractions. (**a**) Cracking moment. (**b**) Ultimate bearing capacity. (**c**) Concrete strain.

As shown in Figure 9, compared with the beam without steel fiber, the cracking and ultimate bending moment of the SFRAC beam increase significantly. When the steel fiber volume fraction is increased to 2%, the ultimate bending moment of the SFRAC beam is increased by 31.86%. As the SF volume fraction increases, the deformation is greater when the beam is damaged. For RC beams with the recycled aggregate replacement rate of 50%, when the steel fiber volume fraction increases to 0.5%, 1.0%, 1.5%, and 2.0%, the cracking moment increases by 1.8%, 12.0%, 12.3%, and 12.6%, respectively. The ultimate bending moment increases by 7.3%, 19.5%, 19.2%, and 24.8%. However, adding 0.5% or more SF into the concrete can offset the effect of RCA on the beam. Figure 9a,b demonstrates that, for beams with 1% or more SF added, the replacement of NCA with RCA has little influence on the beam’s cracking and ultimate bending moments. When the SF content exceeds 1%, the cracking and ultimate bending moments change slightly. When the beam is loaded, the steel fiber alleviates the stress at the tip of the cracks, which delays the concrete’s cracking. As the steel fiber volume fraction increases, the tensile strength of SFRAC is enhanced [32], and the beam cracking moment increases. Under the same condition, the larger the steel fiber volume fraction, the more the steel fibers span the concrete crack in the tension zone, the greater the tensile force, and the larger the ultimate bending moment of the SFRAC beam. Figure 9c shows the stress–strain relationship of the RC beam load with different steel fiber volume fractions when the recycled aggregate replacement rate is 50%. It can be found that the ultimate compressive strain corresponding to the ultimate load can be increased by adding steel fiber. After adding steel fiber, the strain when the recycled concrete is crushed increases from 0.0028 (without steel fiber) to 0.0046 (steel fiber volume fraction of 2%).

## 4. Calculation of Cracking Moment and Ultimate Bending Moment

There is no specification for the steel fiber recycled concrete beam design or the calculation of the cracking moment and ultimate bending moment. In order to study the applicability of the formula in the existing specification to the SFRAC beam, the typical working conditions in the research and the analytical formula are compared.

**Figure 10:**
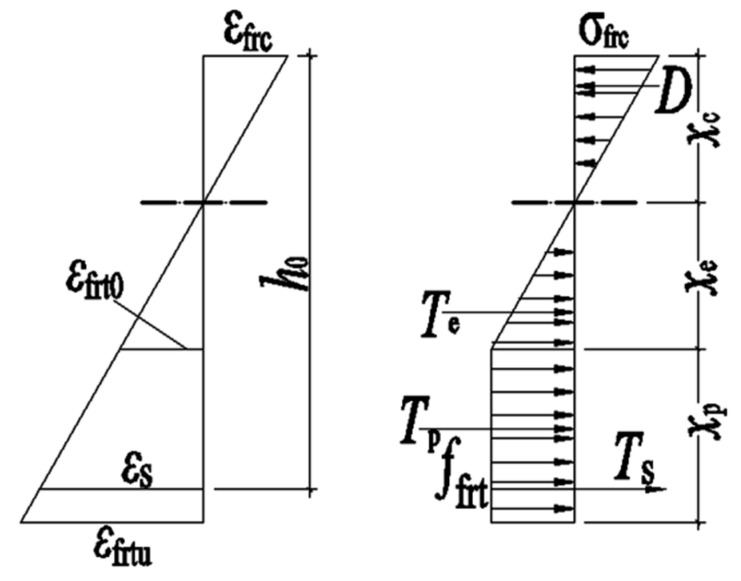
Calculation model of crack resistance of normal section.

The test beam’s cracking moment is calculated using ACI 544.4R-88 [35] and ACI 318-14 [36]. The typical stress–strain distribution of the SFRAC beam is shown in Figure 10.

ACI 544.4R-88 [35] and ACI 318-14 [36] are used to calculate the ultimate bending moment of the test beam. The bending moment distribution in the limit state is shown in Figure 10.

In order to establish a calculation model of crack resistance and the bearing capacity of the normal section of the SFRAC beam, the elastic–plastic steel stress–strain relationship model is used based on the assumption of the flat section. $\sigma_{s}=E_{s}A_{s}\leq f_{y}$; the ultimate tensile strain of the longitudinal tensile steel bar is 0.01. The compressive stress–strain relationship of SFRAC in [37] is adopted, and the contribution of SFRAC at the tensile part of the beam section is considered.

### 4.1. Calculation Method of the SFRAC Beam’s Cracking Moment

The test results demonstrate that the compression zone of SFRAC is in the elastic phase when the steel SFRAC beam is about to crack, and the stress distribution is approximately triangular. The stress distribution in the tension zone is a curve, which can be simplified into a trapezoidal distribution, as shown in Figure 10. According to the fitting analysis of the experimental data, the calculation formula is as below:

(1) $$f_{\mathrm{frt}}=f_{t}\left(1-0.1848\delta_{R}\right)\left(1+0.4041\lambda_{f}\right)$$

From the force equilibrium condition *D* = *T*_e_ + *T*_p_ + *T* in Figure 10, the following can be found:

(2) $$\frac{f_{\mathrm{frt}}bx_{c}^{2}}{2\left(1-\mu \right)\left(h-x_{c}\right)}=\frac{1}{2}f_{\mathrm{frt}}b\left(1-\mu \right)\left(h-x_{c}\right)+f_{\mathrm{frt}}b\mu \left(h-x_{c}\right)+\frac{\alpha_{E}f_{\mathrm{frt}}\left(h_{0}-x_{c}\right)A_{s}}{\left(1-\mu \right)\left(h-x_{c}\right)}$$

According to the moment balance of *D*, *T*_e_, *T*_p_, and *T*_s_ on the neutral axis, the following can be found:

(3) $$M_{\mathrm{frcr}}=f_{\mathrm{frt}}W_{\mathrm{frc}}$$

(4) $$W_{\mathrm{frc}}=\frac{bx_{c}^{3}}{3\left(1-\mu \right)\left(h-x_{c}\right)}+\frac{1}{3}b{\left(1-\mu \right)}^{2}{\left(h-x_{c}\right)}^{2}+b\mu {\left(h-x_{c}\right)}^{2}\left(1-\frac{\mu }{2}\right)+\frac{\alpha_{E}{\left(h_{0}-x_{c}\right)}^{2}A_{s}}{\left(1-\mu \right)\left(h-x_{c}\right)}$$

In order to simplify the calculation, the resistance moment plasticity coefficient $\gamma_{\mathrm{frm}}$ of the SFRAC section is introduced to calculate the cracking moment according to the material mechanics method, i.e.:

(5) $$M_{\mathrm{frcr}}=\gamma_{\mathrm{frm}}f_{\mathrm{frt}}W_{\mathrm{fr}0}$$

(6) $$W_{\mathrm{fr}0}=\frac{\left[bh^{3}+3bh{\left(2x_{c}-h\right)}^{2}+12\left(\alpha_{E}-1\right){\left(h_{0}-x_{c}\right)}^{2}A_{s}\right]}{12\left(h-x_{c}\right)}$$

Simultaneous Formulas (3) and (5):

(7) $$\gamma_{\mathrm{frm}}=W_{\mathrm{frc}}/W_{\mathrm{fr}0}$$

**Figure 11:**
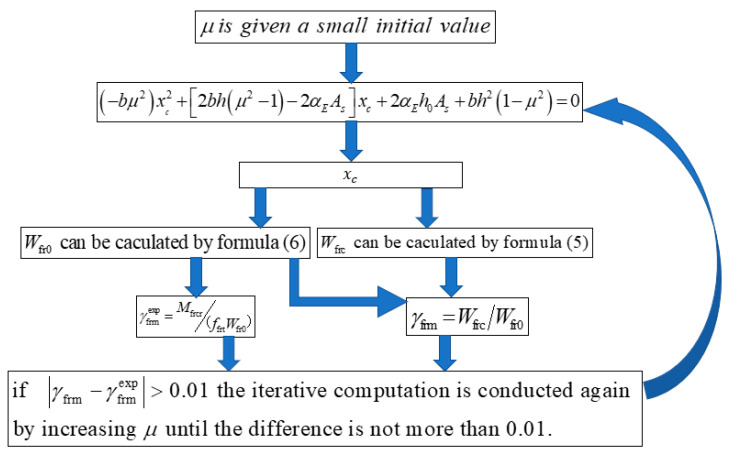
Procedure to calculate *γ*_rfm_.

The procedure to calculate *γ*_rfm_ is shown in Figure 11. For a given SFRAC beam, *b*, *h*, *A*_s_, and *α*_E_ are known. When *μ* is obtained, *x*_c_, $M_{\mathrm{frcr}}$, and $\gamma_{\mathrm{frm}}$ can be calculated. $\gamma_{\mathrm{frm}}^{\exp}$ can be obtained according to Formula (4). As shown in Figure 11, *μ* is given a small initial value, and then $\gamma_{\mathrm{frm}}$ and $\gamma_{\mathrm{frm}}^{\exp}$ are obtained. If the difference between $\gamma_{\mathrm{frm}}$ and $\gamma_{\mathrm{frm}}^{\exp}$ is greater than 0.01, the iterative computation is conducted again by increasing *μ* until the difference is less than 0.01. The calculation is terminated, and the final value of *μ* is obtained. The calculation process is cumbersome, and the calculation program is compiled. The calculation and analysis results of the test data are listed in Table 5. It can be found that the height of the plastic zone of the SFRAC beam is about 0.31~0.56 times the height of the entire tension zone.

As shown in Table 5, the plastic coefficient *γ*_frm_ of the SFRAC beam is larger than that of the ordinary concrete beam. Therefore, the effects of the recycled aggregate replacement rate and the steel fiber characteristic content on the SFRAC beam can be considered by introducing the crack resistance influence coefficients *α*_2_ and *β*_2_ based on 7.2.4 in the *Concrete Structure Design Specification* (GB 50010-2010) [38];

(8) $$\gamma_{\mathrm{frm}}=\left(1+\alpha_{2}\delta_{R}+\beta_{2}\lambda_{f}\right)\left(0.7+120/h\right)\gamma_{m}$$

where *h* and *γ_m_* are consistent with the specification [38] and α_2_ and β_2_ are cracking resistance influence coefficients. The test data are conducted with fitting analysis; *α*_2_ = −0.12 and *β*_2_ = 0.29.

The mean value, mean square, and coefficient of variation of the measured value $\gamma_{\mathrm{frm}}^{\exp}$ and the calculated value with Formula (8) are 0.98, 0.17, and 0.18, respectively. Formula (8) is substituted into Formula (5) to obtain the calculated value of the cracking moment, as shown in Table 5. The mean value, mean square, and coefficient of variation of the ratio of the measured cracking moment and the calculated value are 0.98, 0.17, and 0.19, respectively. The calculated values are consistent with the experimental values. The mean value of the resistance moment plastic influence coefficient $\gamma_{\mathrm{frm}}$ of the SFRAC beam section is 1.59. The resistance plasticity influence coefficients of ordinary concrete and recycled concrete beams are 1.55 [38] and 1.338 [32], respectively. The greater the influence coefficient of the section resistance moment, the better the crack resistance, indicating that adding steel fiber into recycled concrete can compensate for the deterioration of crack resistance caused by the recycled aggregate, improve the crack resistance of recycled concrete, and cause the SFRAC beam to have better crack resistance than ordinary concrete beams.

**Figure 12:**
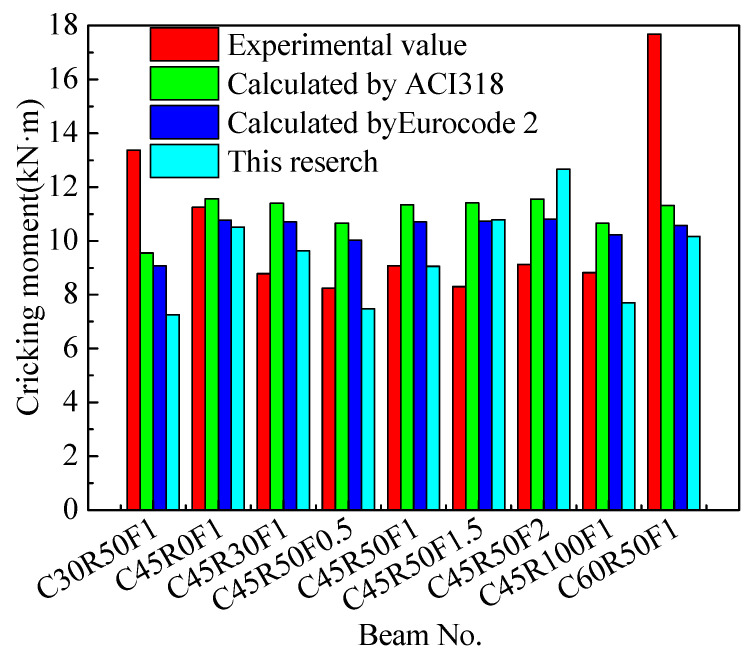
Cracking moments calculated with ACI318 [36], Eurocode [39], and this research.

ACI 318 [36] and Eurocode 2 [39] provide formulas for calculating cracking moments. The calculated values are shown in Figure 12. It can be seen that the calculated values of the cracking moment of beams based on ACI 318 [36] and Eurocode 2 [39] are greater than the experimental values except for beams C30R50F1, C60R50F1, and C45R0F1. The method presented in this paper agrees with the experimental results. Except for beams C30R50F1 and C60R50F1, the cracking bending moments calculated using Formula (3) are close to the test results. However, there are few experimental data that need further research.

### 4.2. Calculation Method for the Flexural Bearing Capacity of the SFRAC Beam

**Figure 13:**
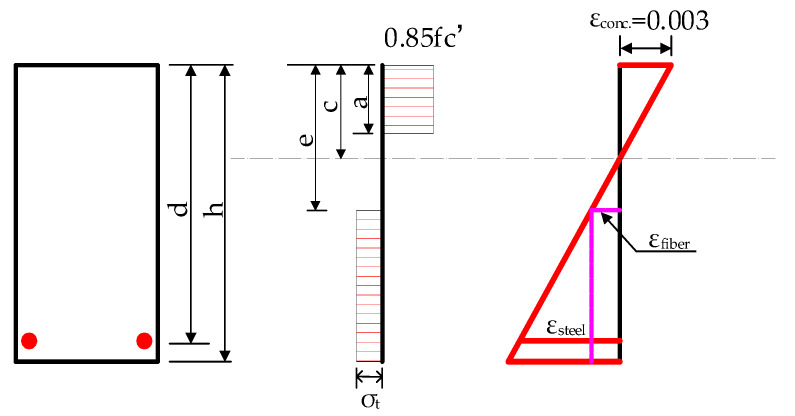
Normal section stress distribution of normally reinforced concrete beam at ultimate state.

The normal section stress distribution of the normally reinforced concrete beam at its ultimate state is shown in Figure 13. The ultimate bending moment of the SFRAC beam is calculated by the equations of ACI544.4R-88 [35] shown in Formulas (9)–(12) to study the effect of the SF volume fraction on the ultimate bending moment of the SFRAC beam:

(9) $$M_{a}=f_{y}A_{s}\left(d-\frac{a}{2}\right)+\sigma_{t}b\left(h-e\right)\left(\frac{h}{2}+\frac{e}{2}+\frac{a}{2}\right)$$

(10) $$a=\frac{\sigma_{t}b\left(h-e\right)+f_{y}A_{s}}{0.85f_{c}^{\prime }b}$$

(11) $$e=\left[\varepsilon_{f}+0.003\right]c/0.003$$

(12) $$\sigma_{t}=0.00772l_{f}/d_{f}V_{f}F_{\mathrm{be}}$$

The ultimate bending moment without SF beam is calculated by using ACI 318-2014 [36]:

(13) $$a=\frac{f_{y}A_{s}}{0.85f_{c}^{\prime }b}$$

(14) $$M_{u}=f_{y}A_{s}\left(d-\frac{a}{2}\right)$$

The theoretically calculated values and the measured values of the ultimate bending moments of all test beams are compared, as shown in Table 6. The mean value, standard deviation, and coefficient of variation of the ratio of $M_{\mathrm{fru}}^{c}$ to $M_{\mathrm{fru}}^{\exp}$ are 0.92, 0.10, and 0.12. Furthermore, $M_{\mathrm{fru}}^{c}$ is the calculated value of the ultimate bending moment, and $M_{\mathrm{fru}}^{\exp}$ is the experiment value of the ultimate bending moment.

**Figure 14:**
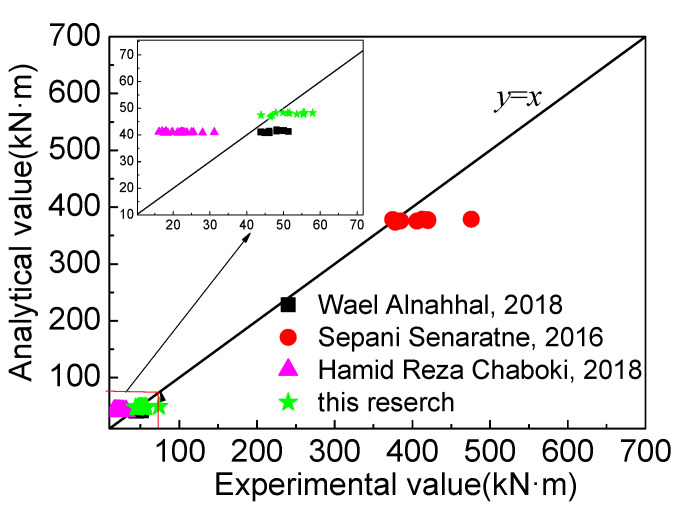
Comparison of calculated results [20,26,33] and test data.

The calculation results of the data in the existing literature are shown in Figure 14. It is obvious that the test deviation of Hamid Reza Chaboki [26] is relatively large, and the section is a shallow beam with a size of 150 mm × 300 mm. The calculation value is relatively large, and the other values are in agreement. The mean value of the ratio of the calculated value to the measured value is 0.90, the mean square error is 0.07, and the coefficient of variation is 0.08.

## 5. Conclusions

This study focused on the bending performance of SFRAC beams under different water–cement ratios, recycled aggregate replacement rates, and steel fiber volume fractions. The main conclusions are as follows:

(1) The cracking and ultimate moment of SFRAC beams are higher than those of ordinary concrete reference beams.
(2) The cracking and ultimate moment of SFRAC beams increase with the decrease in the water–cement ratio. The water–cement ratio decreases from 0.55 to 0.3, and the cracking moment and ultimate moment of SFRAC beams increase by 32.2% and 7.6%, respectively.
(3) The cracking and ultimate bending moment of SFRAC beams are significantly higher than those of reference beams, and the cracking and ultimate moment of SFRAC beams increase with the increase in the steel fiber volume ratio. Compared with recycled concrete beams without steel fiber, the cracking moment and ultimate moment of SFRAC beams are increased by 12.6% and 24.8%, respectively, when the volume fraction of the steel fiber is 2.0%.
(4) After adding recycled aggregate, the cracking moment and ultimate load of steel-fiber-reinforced concrete beams decrease, but the replacement rate of recycled aggregate has little effect on the cracking moment and ultimate load of SFRAC beams. When the replacement rate of recycled aggregate is increased from 30% to 100%, the cracking moment of the SFRAC beam is increased by 0.6%, and the ultimate moment is decreased by 4.7%.
(5) Considering the influence of the recycled aggregate replacement rate and fiber volume ratio, the calculation formula of the cracking moment of SFRAC beams is established, and the prediction accuracy is better than ACI318 and Eurocode 2.
(6) The calculated values of the ultimate bending moment bearing capacity based on ACI 318 and ACI 544 agree with the experimental values, which can reasonably predict the bending bearing capacity of SFRAC beams.
(7) Steel fiber recycled concrete is a material with good performance, and the performance of SFRAC beams with 100% recycled aggregate replacement rate is still higher than that of ordinary concrete benchmark beams, so SFRAC can be used for general load-bearing beam structural members. Applying SFRAC to structural members increases the resource utilization of construction waste and meets the requirements of sustainable development and the transition to green building materials and technologies. In addition, the fatigue performance of SFRAC beams is also a point worthy of further study to expand its engineering application scope.

## Figures and Tables

**Table 1:** The physical properties of cement.

Standard Consistency (%)	Fineness (%)	Specific Surface Area (m^2^/kg)	Density (kg/m^3^)	Loss on Ignition of SO^3^ (%)	Stability
28.5	4.7	3460	3043	2.3	Qualified
Setting time (min)		Compressive strength (MPa)		Flexural strength (MPa)	
Initial setting	Final setting	3 d	28 d	3 d	28 d
142	229	26.1	49.4	4.97	8.64

**Table 2:** The physical properties of coarse aggregate.

Aggregate	Apparent Density (kg/m^3^)	Bulk Density (kg/m^3^)	Water Absorption (%)	Acicular Content (%)	Mud Content (%)	Porosity (%)	Crush Index (%)
RCA	2660	1410	3.74	1.4	0.423	47	13.5
NCA	2730	1360	0.6	3.2	0.925	40	12.0

**Table 3:** Design of mixture proportion.

Beam ID	Water/Cement	RCA Replacement (%)	Steel Fiber Content (%)	Water (kg/m^3^)	Cement (kg/m^3^)	River Sand (kg/m^3^)	RCA (kg/m^3^)	NCA (kg/m^3^)
C30R0F0	0.55	0	0	166	302	884	0	1080
C45R0F0	0.4	0	0	166	415	839	0	1024
C60R0F0	0.3	0	0	166	553	783	0	958
C45R0F1	0.4	0	1	166	415	839	0	1024
C45R30F1	0.4	30	1	166	415	839	307	717
C45R50F1	0.4	50	1	166	415	839	512	512
C45R100F1	0.4	100	1	166	415	839	1024	0
C45R50F0	0.4	50	0	166	415	839	512	512
C45R50F0.5	0.4	50	0.5	166	415	839	512	512
C45R50F1.5	0.4	50	1.5	166	415	839	512	512
C45R50F2	0.4	50	2	166	415	839	512	512
C30R50F1	0.55	50	1	166	302	884	540	540
C60R50F1	0.3	50	1	166	553	783	479	479

**Table 4:** Test results of beams.

Beam ID	$f_{\mathrm{frc}}$	$f_{\mathrm{frts}}$	$E_{\mathrm{frc}}$	Cracking Moment (kN)	Ultimate Load (kN)
	MPa	MPa	×10^4^ MPa		
C30R0F0	36.18	1.92	3	12.5	104.3
C45R0F0	45.68	2.49	3.59	18.2	97.6
C60R0F0	58.43	2.88	3.6	16.2	106.5
C45R0F1	47.69	5.34	3.64	17.6	164.7
C45R30F1	46.39	5.75	3.45	19.5	119.3
C45R50F1	45.88	4.81	3.3	20.2	123.3
C45R100F1	40.5	4.68	2.97	19.6	113.7
C45R50F0	37.26	1.99	3.13	18	103.2
C45R50F0.5	40.58	3.45	3.42	18.3	110.7
C45R50F1.5	46.44	6.14	3.38	18.5	122.9
C45R50F2	47.62	7.18	3.55	20.3	128.7
C30R50F1	32.58	4.39	3.16	13.4	114.8
C60R50F1	45.74	4.22	3.38	17.7	123.5

**Table 5:** Test data and calculated data of cracking moments.

Beam No.	$M_{\mathrm{frcr}}^{\exp}$ (kN·m)	*μ*	$\gamma_{\mathrm{frm}}^{\exp}$	$\gamma_{\mathrm{frm}}$	$M_{\mathrm{frcr}}^{c}$ (kN·m)
C30R0F0	5.63	0.43	1.54	1.55	5.67
C45R0F0	8.20	0.58	1.80	1.55	7.08
C60R0F0	7.29	0.35	1.42	1.55	7.93
C45R0F1	11.25	0.66	1.98	1.83	10.51
C45R30F1	8.78	0.48	1.63	1.78	9.63
C45R50F1	9.08	0.55	1.74	1.74	9.06
C45R100F1	8.82	0.59	1.86	1.65	7.70
C45R50F0	8.10	0.63	1.93	1.46	6.04
C45R50F0.5	8.25	0.56	1.77	1.60	7.48
C45R50F1.5	8.30	0.37	1.45	1.88	10.79
C45R50F2	9.13	0.38	1.47	2.02	12.67
C30R50F1	6.02	0.36	1.44	1.74	7.25
C60R50F1	7.96	0.31	1.37	1.74	10.17

**Table 6:** Ultimate bearing capacity calculated data with test data of SFRAC beams.

Beam No.	$M_{u}^{c}$	$M_{u}^{\exp}$	$\frac{M_{u}^{c}}{M_{u}^{\exp}}$
C30R0F0	47.3	46.9	1.0
C45R0F0	48.2	51.7	0.9
C45R50F0	47.4	43.9	1.1
C60R0F0	49.0	74.1	0.7
C45R50F0.5	47.8	53.7	0.9
C30R50F1	46.8	46.4	1.0
C45R0F1	48.4	49.8	1.0
C45R100F1	47.8	55.5	0.9
C45R30F1	48.3	55.3	0.9
C45R50F1	48.3	57.9	0.8
C60R50F1	48.3	51.2	0.9
C45R50F1.5	48.3	47.9	1.0
C45R50F2	48.4	55.6	0.9

## Data Availability

Data requirements can be directed to the corresponding author.

## Abbreviations

$\sigma_{s}$	Reinforcement stress (MPa)
$E_{s}$	Elastic modulus of steel bar (MPa)
*x*_c_	Height of the compression zone (mm)
*x*_e_	Height of the elastic part of the tension zone (mm)
*x*_p_	Height of the elastic part of the plastic part (mm)
*μ*	Ratio of the height of the plastic zone to the height of the tension zone, *μ = x*_p_/(*h* − *x*_c_);
$\varepsilon_{\mathrm{frc}}$	Ultimate compressive strain of SFRAC (με)
$\sigma_{\mathrm{frc}}$	Ultimate compressive stress of SFRAC (με)
$\varepsilon_{\mathrm{frt}0}$	Peak tensile strain (με)
$\varepsilon_{\mathrm{frtu}}$	Ultimate tensile strain of SFRAC (με)
*D*	Resultant force of the compression zone (N)
*T*_e_	Resultant force of the elastic zone in the tension zone. (N)
*T*_p_	Resultant force of the plastic region zone in the tensile region (N)
*T*_s_	Resultant force of the longitudinal tensile bar (N)
$\delta_{R}$	Recycled Aggregate Replacement Rate
$\lambda_{f}$	Steel Fiber Volume Fraction
*f*_frt_	Tensile strength of SFRAC (N)
*f*_y_	Designed strength of the steel bar (MPa)
*f*_t_	Tensile strength of ordinary concrete with the same SFRAC strength grade (MPa)
*W*_fr0_	Resistance moment of the converted section after the longitudinal tensile reinforcement is converted into the SFRAC area.
*b*	Width of the beam section (mm)
*h*	Height of the beam section (mm)
*h*_0_	Effective height of the beam section (mm)
$\alpha_{E}$	Ratio of the elastic modulus of the longitudinal tensile steel bar to the SFRAC elastic modulus
$\gamma_{\mathrm{frm}}^{\exp}$	Measured value of the resistance moment plastic influence coefficient of the SFRAC section
$M_{\mathrm{frcr}}^{\exp}$	Measured cracking moment (kN·m)
$M_{\mathrm{frcr}}^{c}$	Calculation value of cracking moment (kN·m)
*M*_frcr_	Cracking moment of the SFRAC beam (kN·m)
$\gamma_{\mathrm{frm}}^{}$	Resistance moment plastic influence coefficient of the SFRAC section
*W*_fr0_	Resistance moment of the converted section after the longitudinal tensile reinforcement is converted into the SFRAC area (mm^3^)
*W*_frc_	Elastoplastic resistance moment of the section of the SFRAC beam to the tension zone edge of the section considering the plastic deformation (mm^3^)
$M_{a}$	Ultimate bending moment of SFRAC beam is calculated according to ACI 544.4 R-88
$M_{u}$	Ultimate bending moment of SFRAC beam is calculated according to ACI 318-2014
*d*	Distance from extreme compression fiber to centroid oftension reinforcement (mm)
*a*	Depth of rectangular stress block b =width of beam (mm)
*c*	Distance from extreme compression fiber to neutral axis found byequating the internal tension (mm)
*e*	Distance from extreme compression fiber to top of tensile stress block of fibrous concrete (mm)
$f_{c}^{\prime }$	Compressive strength of concrete (MPa)
$\sigma_{t}$	Tensile stress in fibrous concrete (MPa)
$F_{\mathrm{be}}$	Bond efficiency of the fiber
$\varepsilon_{f}$	Tensile strain in steel fiber at theoretical moment strength of beam
$M_{{}_{u}}^{c}$	Theoretically calculated value of the ultimate bending moment (kN·m)
$M_{{}_{u}}^{\exp}$	Measured value of the ultimate bearing capacity (kN·m)
*A_s_*	Section area of the longitudinal tensile bar (mm^2^)
$V_{f}$	Percent by volume of steel fibers
